# Timing the Retirement

**DOI:** 10.1055/s-0045-1815713

**Published:** 2026-02-15

**Authors:** Surajit Bhattacharya

**Affiliations:** 1 Department of Plastic, Reconstructive, and Aesthetic Surgery, Ajanta Hospital, Lucknow, Uttar Pradesh, India

Timing one's retirement is both a tricky and a very personal matter. We published an article on this subject in the *Indian Journal of Surgery* 1 in which we have described how a surgeon should be planning his/her retirement, but again this is what we, the authors of this article, think, and others may have an equally convincing but different opinion. We have also argued against the idea that competition and fear of litigation can drive one into retirement. 2 But, whatever the reason, the fact that this is a watershed moment in a professional's life cannot be denied.

Four retirements come to my mind immediately, one hypermature, Mr. Warren Buffet; two just right, Rafael Nadal and Rohit Sharma; and one immature, Virat Kohli. Clearly, age has nothing to do with this timing because Kohli is just 34, and Warren is older than the S&P 500 (Standard & Poor's 500) and perhaps younger only to God. For CEOs, business leaders, and management gurus, if they cannot climb stairs to reach their corner office when the lift is out of order, God is hinting to them it is time to “let go.” Physical fitness should not be confused with physical handicap, as many specially abled people routinely outperform able-bodied colleagues. When one is not able to give his/her best to a job that has been his/her passion, one should seriously question his/her continuity. If one is a noble personified, like Ratan Tata (of the Tata group), or has a successful succession plan like Azim Premji (of Wipro), one can walk out without hesitation as soon as they stop enjoying what they do. If one is a Mahendra Singh Dhoni or Indra Nooyi (of Pepsi), he/she will stay just long enough to mentor his/her successor, leaving not a void but a blueprint.

So, what exactly makes a professional call it a day is not written in stone. It is a call that comes from within. However, if one's learning curve is steeper than one's age graph, if one's curiosity still outweighs one's ego, and if one can say “I don't know, I'll have to check up” without batting one's eyelid and feeling defensive, one still has fire in one's belly to steam one ahead.

There is another way of knowing if one still has fuel left in the tank. If one is still thinking “how can I contribute” instead of “how can I control” one is still valuable for the profession. If work still makes one happy and not exhausted, if one is still learning and growing and not sulking and stagnating, then this isn't work, but this is purpose. One can retire from work, but purpose never retires.
